# Supplementary material for: Healthy homes: Stakeholder perspectives on housing interventions to reduce environmentally mediated infections
Source: PLOS Glob Public Health. 2025 Apr 21;5(4):e0003805. doi: 10.1371/journal.pgph.0003805 (PMC12011249; doi:10.1371/journal.pgph.0003805)
Supplement: S1 Text — (PDF) [file pgph.0003805.s001.pdf]

Supplementary Material

Supplementary Material 1 – Semi-structured interview guides

Research/Implementation:

- First of all, could you tell me what led you to do research on this topic?

- Could you please tell me about the kinds of housing interventions that you have researched?

- What is/was the goal/outcome of interest of the intervention?

- What has your research found about the impact of housing interventions on health?
    - [Follow-up] Are there other ways you think that housing interventions can potentially influence human health and wellbeing?

- Are there any other interventions you have not yet studied that you think would be impactful?
    - [Follow-up]: Why haven't you studied them?
- What were the motivational drivers for you to research this intervention?
    - [Follow-up] What do you perceive to be the biggest motivational drivers for other stakeholders of the intervention [e.g., funder, or recipient community, government]?
    - [Follow-up] How did you perceive the willingness of the community to implement the intervention?
- Which factors influenced the implementation of the intervention?

Let's now talk about the funding landscape of housing interventions. Some interventions that can potentially have multiple impacts, like housing interventions, might not be as easy to get funded and scaled-up, compared to disease-specific interventions [e.g., medical treatments, such as deworming tablets]

- What is your perspective on that observation, and what might be reasons in your opinion for the preference for such disease-specific interventions?

- What do you think is important to increase awareness/attractiveness and eventually securing funding for such housing interventions?

- With housing interventions you're familiar with, what is the average cost per household?
    - [Follow-up] Do these costs include the transportation of resources, or do they only include production?
    - [Follow-up] How does cost come into play for scale-up?

- Can you tell me about the likelihood that such housing interventions could be scaled-up to reach a larger population?
    - [Follow-up] Who would scale-up the intervention?

- How can/should contextual factors be addressed when thinking about scaling up?

That concludes our questions, but if you have any further thoughts or feel that we have overlooked important aspects, please feel free to share them now.

Policy:

- Could you please tell me about the kinds of housing interventions that you have delivered and/or scaled up?

- What were the primary motivations behind delivering/scaling up this housing upgrade intervention?

- Can you outline the key policy drivers that influenced the decision to implement this intervention on a larger scale?

    - [Follow-up]: Were there any specific economic, social, or environmental objectives driving the decision to scale up this intervention?

- How did the local or national housing policies inform the design, implementation, and if applicable, scale up of this intervention?

- What were the main challenges or barriers encountered in delivering and/or scaling up the intervention, particularly in relation to policy implementation?

- How were stakeholders, such as government agencies, local communities, and NGOs, involved in shaping the policy direction of the housing upgrade intervention?

- Can you discuss any data or evidence that supported the decision to scale up this intervention?

    - [Follow-up]: How was this evidence used in the policy-making process?

- Did the intervention align with any broader development goals or strategies set by the World Bank or other international organizations?

- How did considerations of equity and social justice influence the decision-making process regarding the scaling up of the housing upgrade intervention?

- Looking ahead, what are the anticipated long-term impacts of scaling up this intervention, both in terms of policy outcomes and broader development goals?

That concludes our questions, but if you have any further thoughts or feel that we have overlooked important aspects, please feel free to share them now.
