# Supplementary material for: Healthy homes: Stakeholder perspectives on housing interventions to reduce environmentally mediated infections
Source: PLOS Glob Public Health. 2025 Apr 21;5(4):e0003805. doi: 10.1371/journal.pgph.0003805 (PMC12011249; doi:10.1371/journal.pgph.0003805)
Supplement: Attachment — Submitted filename: Comments - From the ground up.docx [file pgph.0003805.s003.docx]

**Comments:**

This is a very well-written manuscript, on a critical topic and I enjoyed reading it.

1. I fail to connect the first part of the title and the manuscript, “From the ground up”, I think the content is mostly about the Stake holders perspectives on housing intervention to reduce diseases and nothing substantial about the bottom – up approach.
2. I think in the background literature, we are missing information regarding ventilation in relation to indoor air pollution and respiratory tract infections which is one of the environmentally mediated infections
3. I can see in the results section you have used words like “several KIs” or “Many KIs”. I recommend providing information about the density of that information. How many KIs mentioned it (for example 6/16), this provide a bit of insight of the depth of this information.
