# Supplementary material for: Healthy homes: Stakeholder perspectives on housing interventions to reduce environmentally mediated infections
Source: PLOS Glob Public Health. 2025 Apr 21;5(4):e0003805. doi: 10.1371/journal.pgph.0003805 (PMC12011249; doi:10.1371/journal.pgph.0003805)
Supplement: Attachment — Submitted filename: Manuscript Reviews_rev.docx [file pgph.0003805.s004.docx]

**Manuscript Reviews:**

**Reviewer #1:**

**Abstract:**

This does not set up the paper very well. It cites an example of housing improvements as being 'finished floors', which is not the example I was expecting (such as running water, heating and cooling, crowding levels etc).

- Thank you for this feedback. We agree that some elements of housing improvements considered in this manuscript are limited. For example, there has been a long history of research focused on household air pollution from the use of solid fuels for cooking and heating, as well as household water and sanitation interventions. Each of these two research areas has a large body of research dedicated to the topic. We chose not to focus on water and sanitation or indoor air pollution improvements due to the large number of studies conducted on these topics. We instead aimed to focus on less-recognized hazards, such as flooring that you noted, where infants and children spend a large amount of time. We have, however, made revisions to better expand upon how we define housing improvements in the abstract.

In the abstract Background, we revised as follows: “*Beyond efforts to improve water and sanitation and reduce household air pollution, housing improvements remain relatively under-explored as health interventions.”*

In the abstract Methods, we revised as follows: “*Sixteen key informants (KIs) with direct experience conducting housing interventions with a goal to reduce environmentally mediated infectious diseases in low- and middle-income countries were interviewed using a semi-structured interview format.”*

The results summary is very broad when details would have been helpful.

- Thank you, we have pulled out more specific results to address this point.

'Infections' are mentioned broadly, but details (e.g., infection names or groups) would have been beneficial.

- We have added additional details about the environmental mediated infections that were the focus of this analysis.

Overall, the abstract lacks details about the study- which is then reflected in the body of the paper.

- Given word limits, there was limited space to add detail in the abstract, but we have revised the manuscript to address this critique and have now added additional detail. Fo example, see additions to Table 1, details about our analytic method starting on line 206, and additional discussion of limitations starting on line 616.

  **Background:**

The scope of the research is extremely broad; there is insufficient detail on the countries / regions of focus and on the issues of concern (e.g., transmission of infections); there is a focus on finished floors and vector borne infections but these two areas do not have a logical link to each other; climate change issues are raised without sufficient connection to housing... in sum, the scope is extremely broad and prevents tangible results being identified that can then be implemented into housing developments of policy.

- We agree that the scope of this study was broad. This was done purposely because in our review of the literature, we found very few studies on housing interventions in LMICs that aimed to reduce infectious diseases – with the exception of water, sanitation and hygiene, household air pollution and bednet interventions.

  **Methods**

The sample size (16 key informants) was very small. This can be justified, though, if the authors wish to do so.

- The sample size was limited by the number of key informants we could identify who have worked on housing interventions aimed at reducing infectious diseases. Additionally, we asked the key informants for additional contacts of people who may have knowledge of housing interventions that aim to reduce environmentally mediated infectious diseases. We have noted in the manuscript that the sample size was a limitation to the study.

We added the following starting on line 616: *“There were several limitations of this study. The sample size was limited by the number of key informants we could identify who have worked on housing interventions aimed at reducing infectious diseases; the small number of stakeholders may have restricted the viewpoints and themes identified in the analysis. For example, certain barriers such as lack of land ownership or the limitations faced by renters, as well as household crowding were not raised in the interviews.”*

The key informants are said to describe low- and middle-income countries. Can more detail please be provided regarding the countries about which or from which the informants were reporting? This may enable reviewers to understand whether this is a strong sample from which findings can be assessed as robust.

- Yes, we added the primary country/region of relevant work experience about which countries the key informants were reporting on (see Table 1).

**Discussion:**
Some literature is referred to, but it would be better to return to this literature once it has been cited earlier (e.g., in the Background).

- We have now added additional content into the Background section. On lines 112-122, we added the following: “*Infants and young children are especially vulnerable to unhealthy housing and crowding because they spend most of their time at home [11]. Housing interventions that reduce environmental infections are crucial for protecting their health. Pharmaceutical treatments often do not address environmental pathogen reservoirs and thus may not be able to achieve sustained disease transmission reductions on their own [10]. While decades of research have investigated specific housing-related interventions, including: water, sanitation, and hygiene (WASH), household air pollution, and bednets [12–21], other types of housing interventions have been relatively unexplored. Less prominent housing interventions designed to reduce environmentally mediated infectious diseases in low- and middle-income countries can be broadly categorized into two main areas: finished floors and housing upgrades for vector-borne disease prevention (Figure 1).”*

I am intrigued that crowding was only mentioned once but not extrapolated. Crowding has so many impacts on human health in housing settings that this is a key aspect to consider.

- We have added additional content about crowding in the Background and Discussion section, however it was not a theme that was commonly raised by key informants during the in-depth interviews.

On line 88, we revised as follows: “*Housing-related factors that can affect disease risk are 1) building structure, 2) water supply, 3) sanitation, 4) refuse storage and collection, 5) food storage and preparation, 6) cooking and heating fuels and infrastructure, 7) crowding, and 8) diseases associated with the geographical location of the housing [1,8,9].”*

On lines 616-624 we revised as follows: “*The sample size was limited by the number of key informants we could identify who have worked on housing interventions aimed at reducing infectious diseases; the small number of stakeholders may have restricted the viewpoints and themes identified in the analysis. For example, certain barriers such as lack of land ownership or the limitations faced by renters, as well as household crowding were not raised in the interviews.”*

I was hoping that the authors may propose a way to evaluate how health is impacted by housing and housing improvements. It was a shame that this was not raised.

- We recognize that this study will not answer many important questions of interest to the housing and health community, however our goal was to explore facilitators of and barriers to funding, implementing, and scaling up housing improvements as health interventions.

**Reviewer #2:**

This is a very well-written manuscript, on a critical topic and I enjoyed reading it.

I fail to connect the first part of the title and the manuscript, “From the ground up”, I think the content is mostly about the Stake holders perspectives on housing intervention to reduce diseases and nothing substantial about the bottom – up approach.

- Our intention with 'From the Ground Up' was to use a metaphor referring to houses being built literally from the ground up, highlighting the importance of foundational improvements in housing to enhance health outcomes. It wasn’t meant to imply a bottom-up approach.

  We have revised the study title to: “*Healthy Homes: Stakeholder Perspectives on Housing Interventions to Reduce Environmentally Mediated Infections*”

I think in the background literature, we are missing information regarding ventilation in relation to indoor air pollution and respiratory tract infections which is one of the environmentally mediated infections

- This is an important point. There is extensive research on household air pollution from the use of solid fuels for cooking and heating to reduce respiratory infections, as well as household water and sanitation interventions to reduce enteric infections. We chose not to focus on these two areas due to the large number of studies conducted on these types of interventions. We instead aimed to focus on less-recognized housing-related hazards. We have made revisions to clarify the focus of this particular study.

On lines 116-122, we revised as follows: *“While decades of research have investigated specific housing-related interventions, including: water, sanitation, and hygiene (WASH), household air pollution, and bednets [12–21], other types of housing interventions have been relatively unexplored. Less prominent housing interventions designed to reduce environmentally mediated infectious diseases in low- and middle-income countries can be broadly categorized into two main areas: finished floors and housing upgrades for vector-borne disease prevention (Figure 1).”*

I can see in the results section you have used words like “several KIs” or “Many KIs”. I recommend providing information about the density of that information. How many KIs mentioned it (for example 6/16), this provides a bit of insight of the depth of this information.

- Our approach was rooted in reflexive thematic analysis (Braun & Clarke, 2019), which emphasizes identifying patterns and significant insights rather than using the frequency of mentions. While we agree that it can be helpful to provide context about how widely a theme was shared, we chose to prioritize the depth and relevance of themes over counts (e.g. content analysis). Specifically, we followed the approach recommended in the publication, “To saturate or not to saturate? Questioning data saturation as a useful concept for thematic analysis and sample-size rationales” (<https://doi.org/10.1080/2159676X.2019.1704846>) where the authors recommend “information power” as a more useful way of thinking through data samples than frequency of mentions.

We added the following on lines 206-208: *“Our approach was rooted in reflexive thematic analysis [32], which emphasizes identifying patterns and significant insights and prioritized the depth and relevance of themes over counts (e.g., content analysis).”*

- If this reviewer and editors prefer a metric for the frequency of the responses, we are happy to make the changes.

**Reviewer #3:**

Thank you for the opportunity to review this well-executed and insightful manuscript. The study addresses an important and timely topic, with housing interventions holding significant potential for improving health outcomes and advancing Sustainable Development Goals in the face of population growth and climate change. The paper is well-organized, clearly written, and provides valuable perspectives on barriers and facilitators to implementing these interventions in low- and middle-income countries. Additionally, the thematic analysis is robust and offers meaningful insights. However, there are notable gaps in the perspectives included, particularly regarding the diversity of voices represented and the exploration of actionable takeaways. Strengthening these aspects could further enhance the study’s impact and utility for practitioners, policymakers, and researchers alike.

- These are two important critiques, and we have attempted to further expand upon the actionable items that came out of the interviews. We added the following starting on line 588: “*There are likely many important examples that could be used for effectively scaling up housing interventions. A notable one is the Swachh Bharat Mission (Gramin), or ‘Clean India Mission’ program that estimates to have greatly increased sanitation coverage in rural India, estimated to have shifted rural sanitation coverage from 39% to nearly 95% between 2014 to 2019 [36]. Research suggests that the Swachh Bharat Mission, estimated to cost $9.7 billion USD [37], succeeded in-part through committing to ambitious targets, utilizing more modern approaches to messaging, marketing and monitoring, and publicly rewarding and recognizing implementers involved in achieving the programmatic goals [36]. Given the potentially high capital costs for some housing improvements, a combination of grants, loans and tariffs, similar to Swachh Bharat, would likely be required to achieve the level of funding needed for housing improvements at scale. A key takeaway from many other national programs that have been taken to scale is that governments are best positioned to coordinate the implementation of housing programs that will need to be designed to reach the poorest households [38,39].”*
- We added the following starting on line 611: “*Climate change will further exacerbate the need for improved housing [42] and as temperatures increase, the need for housing with better insulation, ventilation, and cooling mechanisms to protect household members will rise. Many health outcomes associated with high temperatures in homes have already been observed, including heat stroke, respiratory and cardiovascular hospitalizations and deaths [43].”*
- To highlight gaps in the diversity of voices represented, we revised the limitations paragraph in the Discussion as follows: “*There were several limitations of this study. The sample size was limited by the number of key informants we could identify who have worked on housing interventions aimed at reducing infectious diseases; the small number of stakeholders may have restricted the viewpoints and themes identified in the analysis. For example, certain barriers such as lack of land ownership or the limitations faced by renters, as well as household crowding were not raised in the interviews. Furthermore, we did not have KIs who represented government or communities; these stakeholders could have provided valuable insights. Engaging community residents involved in housing improvement programs ended up being beyond the scope of this study. Additionally, we sought government-based key informants, however, we were unsuccessful. We were able to interview only one individual who worked closely with the Mexican government during the Piso Firme program. Similarly, we only invited KIs who could complete the interview in English, which further limits the diversity of perspectives in this study. This study did not address the contribution of environmentally related infections outside the home, despite many older children and adults spending much of their time outside the home. Lastly, the broad spectrum of housing interventions was discussed in a generic manner, which overlooks nuances specific to particular interventions.”*

Major:
The manuscript’s title emphasizes "stakeholder perspectives on housing interventions," yet the perspectives presented are predominantly from academics—who may have limited direct involvement in large-scale implementation—and practitioners or policymakers affiliated with large, often multinational organizations. Notably absent are the voices of local or national government officials, despite the frequent identification by respondents as government officials being critical stakeholders in the success or blocking of housing interventions. Similarly, perspectives from grassroots organizations or residents of substandard housing are not included. While the exclusion of residents and grassroots organizations is understandable given the scope of this global study, the absence of government perspectives represents a significant limitation. Clarification regarding whether government officials were contacted but declined to participate, or were intentionally excluded, would be valuable. Addressing this gap or providing a rationale for their absence would strengthen the paper’s comprehensiveness and applicability to real-world policy and implementation.

- We fully agree with this critique. In the early stages of preparing the study, we considered the critical importance of enrolling community residents who had been involved in housing and health-related programs. However, engaging community residents ended up being beyond the scope of what we could do.

  Additionally, we did seek to enroll government-based key informants, however, we were unsuccessful. Our perspective is that many housing and health interventions – besides ones made for sanitation, household energy or bednets – are not a focus of government programs. In the case of sanitation, for example, a lot has been published on the implementation of India's Swachh Bharat Mission–Clean India Campaign that aims to scale basic sanitation across the country, among other interventions.

  The government of Mexico’s *Piso Firme* program was an exception, and we tried to find a government official who was engaged in its implementation. However, in the end, we only were able to interview an individual who worked closely with the Mexican government.

We added the following to the limitations paragraph in the Discussion: “*Furthermore, we did not have KIs who represented government or communities; these stakeholders could have provided valuable insights. Engaging community residents involved in housing improvement programs ended up being beyond the scope of this study. Additionally, we sought government-based key informants, however, we were unsuccessful. We were able to interview only one individual who worked closely with the Mexican government during the Piso Firme program.”*

Two key areas were notably absent from the interviews or their inclusion in the paper, which I found surprising given their potential relevance to the study’s focus. First, the contribution of environmentally related infections outside the home was not addressed, despite the fact that most individuals, particularly in LMICs, spend significant time outside their residences. Exceptions such as young children or the elderly—important at-risk groups—could be acknowledged to better contextualize the risk landscape. Second, barriers to improving housing, including lack of land ownership or the challenges faced by renters, were not discussed. These are critical structural issues that often underpin the feasibility and sustainability of housing interventions and could enrich the discussion. The authors may wish to consider briefly addressing these points in the discussion section. Additionally, it would be helpful to know if there were other themes in the literature that the authors anticipated emerging in interviews but did not. A reflection on these gaps could provide valuable insights into the study’s scope and help situate its findings within broader conversations on housing and health.

- We have now added limitations to this study that include the point about land ownership and renting, which did not come up in our in-depth interviews. We added the following starting on line 616: “*The sample size was limited by the number of key informants we could identify who have worked on housing interventions aimed at reducing infectious diseases; the small number of stakeholders may have restricted the viewpoints and themes identified in the analysis. For example, certain barriers such as lack of land ownership or the limitations faced by renters, as well as household crowding were not raised in the interviews.”*
- We have now better framed the study to emphasize the importance of infants and young children who spend most of their time at home and would benefit the most from these interventions. We added the following starting on line 112: “*Infants and young children are especially vulnerable to unhealthy housing and crowding because they spend most of their time at home [11]. Housing interventions that reduce environmental infections are crucial for protecting their health. Pharmaceutical treatments often do not address environmental pathogen reservoirs and thus may not be able to achieve sustained disease transmission reductions on their own [10]. While decades of research have investigated specific housing-related interventions, including: water, sanitation, and hygiene (WASH), household air pollution, and bednets [12–21], other types of housing interventions have been relatively unexplored. Less prominent housing interventions designed to reduce environmentally mediated infectious diseases in low- and middle-income countries can be broadly categorized into two main areas: finished floors and housing upgrades for vector-borne disease prevention (Figure 1).”*
- However, there was feedback from one of the key informants that noted the importance of protecting people in their homes (at least with malaria and arboviruses) because a large fraction of vectorborne disease transmission occurs indoors at night in the home. Research confirms that nighttime is a critical window of time:
  - Ndenga, B.A., Mutuku, F.M., Ngugi, H.N., Mbakaya, J.O., Mukoko, D., Kitron, U. and LaBeaud, A.D., 2022. Night time extension of *Aedes aegypti* human blood seeking activity. *The American Journal of Tropical Medicine and Hygiene*, 107(1), p.208.
  - Monroe A, Moore S, Koenker H, Lynch M, Ricotta E. Measuring and characterizing night time human behaviour as it relates to residual malaria transmission in sub-Saharan Africa: a review of the published literature. *Malar J*. 2019;18: 6. doi:10.1186/s12936-019-2638-9

We added the following on line 153: “*A relatively large number of studies have also investigated housing improvements that reduce vector entry into the home in order to reduce malaria [24]. Nighttime can be a critical time for protecting household members from vectors [25,26].”*

The authors emphasize the need to gain traction for housing improvements as a strategy to enhance health outcomes. However, evidence from other public health initiatives, such as WASH, indicates that health alone may not be a compelling motivator for action by communities or governments. As noted in the manuscript, housing improvements generate a broad array of co-benefits beyond health, including economic stability, educational advancements, and environmental sustainability. This raises an important question: might advocacy for housing improvements benefit from aligning with campaigns targeting these broader benefits? For example, the introduction connects housing improvements to climate change and WASH interventions, but this link is not revisited in the discussion. Exploring lessons from these fields could strengthen the argument for housing interventions and provide valuable insights into potential advocacy strategies.

- Thank you for highlighting this. We have revised the discussion to include that housing improvements should aim to align with other programs and campaigns to target broader benefits, beyond health. We added the following on line 579: “*Given the need for substantial investment to complete housing upgrades, housing programs may be more successful when aligned with other programs and campaigns to target broader benefits, beyond health.*”
- We also added additional text to the discussion to cover the link of housing to climate change and WASH interventions. We agree that exploring lessons from other fields would strengthen how we conceptualize housing interventions and provide a more strategic path forward. We added the following on line 611: “*Climate change will further exacerbate the need for improved housing [43] and as temperatures increase, the need for housing with better insulation, ventilation, and cooling mechanisms to protect household members will rise. Many health outcomes associated with high temperatures in homes have already been observed, including heat stroke, respiratory and cardiovascular hospitalizations and deaths [44].”*
- On line 568, we state: “*KIs consistently stated that housing interventions should be prioritized within health and development agendas as a complement to existing strategies, such as WASH interventions.”*

Additionally, parallels with WASH suggest that localized improvements (e.g., a few upgraded homes in a slum neighborhood) may not yield measurable health impacts due to continued exposure from the broader environment. Yet, widespread improvements may generate community-level benefits. If this is the case, it underscores the critical role of government in scaling interventions to achieve population-level impacts. The authors could consider discussing whether such collective benefits might strengthen the case for public investment in housing and whether collaboration with sectors like WASH and climate change could amplify advocacy and implementation efforts.

- This is an excellent point and we have now added additional text in the discussion on the importance of government involvement to reach a certain level of saturation with interventions in order to achieve population-level impacts. We added the following on line 598: “*A key takeaway from many other national programs that have been taken to scale is that governments are best positioned to coordinate the implementation of housing programs that will need to be designed to reach the poorest households [39,40].”*

  While the manuscript is very strong overall, the discussion and conclusion sections do not fully match the rigor and depth of the earlier sections. These could be enhanced by addressing the comments raised above and by providing more concrete recommendations on which stakeholders should lead action and the strategies they might employ. Offering actionable insights tailored to different stakeholder groups—such as policymakers, practitioners, and researchers (and ideally government and communities) —would provide a clearer pathway for translating the study’s findings into practice and future research. Additionally, the conclusion could be strengthened by revisiting and emphasizing the stakes involved in not improving housing, as articulated effectively in the introduction.
- We have reworked the discussion to provide more actionable recommendations on which different stakeholders could take action, as well as provided more discussion of potential strategies that could be effective. For example, we added the following on line 595: “*Given the potentially high capital costs for some housing improvements, a combination of grants, loans and tariffs, similar to Swachh Bharat, would likely be required to achieve the level of funding needed for housing improvements at scale. A key takeaway from many other national programs that have been taken to scale is that governments are best positioned to coordinate the implementation of housing programs that will need to be designed to reach the poorest households [39,40].”*

**Minor**

Line 117 – was there anything systematic about the non-response? Were any groups in the original sample under or not represented in the final interviews?

- We have now discussed non-response. We have now highlighted that we were unsuccessful at reaching government stakeholders, which we set out to interview.

Line 154 – suggestion to replace the interviewee’s use of “it” to refer to children with bracketed [they] [he or she] or [the child].

- Edit made.

Line 188 – suggestion to replace the “traditionally used materials” referring to concrete with another word --- “traditional” is used later to describe things like wood. Or if keeping the word traditional, the authors could make it clear which tradition/traditional for whom/as of when.

- Edit made.

Line 265 – I believe “with” is a typo and should be replaced with “for”

- Edit made.

Line 313- suggestion to add the reference for the Piso Firme project (it’s in the intro but readers may have forgotten what the program is)

- Edit made.

Line 376 – it’s unclear to me who the “them” is referring to

- Edit made.

Line 419 -_“tend to over-emphasized economic factors” feels vague – could you provide an example(s).

- Edit made.

Line 511 - suggestion to remove “which may have” and just say “limits the diversity”

- Edit made.

Line 513 - suggestion to remove “may have” and just say “overlooks nuances”

- Edit made.
