# Supplementary material for: Healthy homes: Stakeholder perspectives on housing interventions to reduce environmentally mediated infections
Source: PLOS Glob Public Health. 2025 Apr 21;5(4):e0003805. doi: 10.1371/journal.pgph.0003805 (PMC12011249; doi:10.1371/journal.pgph.0003805)
Supplement: Attachment — Submitted filename: Manuscript Reviews_rev2.docx [file pgph.0003805.s005.docx]

1. I would suggest that they include in the manuscript more of the justification around lack of government representatives as key informants. In their reviewer response (but not in the manuscript) they hypothesize that it was difficult to identify relevant government actors because "housing and health interventions –besides ones made for sanitation, household energy or bednets – are not a focus of government programs." This is an important perspective - perhaps one which they could also support with evidence - as it also has implications for how housing interventions to improve health.

Thank you for this feedback. We added the following new paragraph on lines 537-548 to the discussion, which includes some sentences that were previously in the limitations paragraph as well as additional interpretation and reinforcement from our empirical findings.

*“A limitation of this study is that we were not able to interview any government-based key informants even though we invited them. We were able to interview only one individual who worked closely with the Mexican government during the Piso Firme program. The perspectives of government employees would have shed light on potential barriers to funding, subsidizing, and zoning and permitting processes for housing interventions to improve health. Multiple KIs mentioned that governments would play a critical role in scaling up housing interventions, in part due to their financial resources but also through constitutional mandates in some countries to provide housing for all. Non-response from government-based key informants may reflect a lack of focus on housing and health interventions – besides ones made for sanitation, household energy or bed nets – in government programs. Additionally, KIs remarked that governments may face difficult trade-offs when funding housing interventions, such as choosing between offering tax cuts or reducing spending in other areas.”*

2. Finally, a very minor comment but the section on Swachh Bharat uses the word estimate/estimated many times - I suggest finding some alternative phrasing.

We revised this paragraph as follows on lines 505-511:

*“A notable one is the Swachh Bharat Mission (Gramin), or ‘Clean India Mission’ program that aimed to increase sanitation coverage in rural India, which is estimated to have shifted rural sanitation coverage from 39% to nearly 95% between 2014 to 2019 [37]. Research suggests that the Swachh Bharat Mission, which cost approximately $9.7 billion USD [38], succeeded in-part through committing to ambitious targets, utilizing more modern approaches to messaging, marketing and monitoring, and publicly rewarding and recognizing implementers involved in achieving the programmatic goals [37].”*
